# Case report: First isolation of *Yersinia pseudotuberculosis* from the blood of a cat

**DOI:** 10.3389/fvets.2023.1261925

**Published:** 2024-01-05

**Authors:** Raffaele Scarpellini, Massimo Giunti, Cecilia Bulgarelli, Elisabetta Mondo, Erika Esposito, Giammarco Assirelli, Silvia Piva

**Affiliations:** Department of Veterinary Medical Sciences, University of Bologna, Bologna, Italy

**Keywords:** *Yersinia pseudotuberculosis*, bloodstream infection, feline, bacterial, blood, yersiniosis

## Abstract

A 14-year-old female domestic short-haired cat with a diagnosed diabetes mellitus and acromegaly was presented for lethargy and dysorexia. On clinical presentation, the patient showed hyperglycemia, hyperthermia, dull mentation, and dehydration. With the suspicion of an inflammatory or infectious complication of diabetes, she was hospitalized with constant rate infusion of insulin, and empirical ampicillin sulbactam was started. Blood culture revealed positivity for *Yersinia pseudotuberculosis* and the septic picture was confirmed by blood analysis, with leukocytosis, neutrophilia, and an increased serum amyloid A concentration. The isolated *Y. pseudotuberculosis* strain showed susceptibility to every antimicrobial tested. During the second day of hospitalization, the onset of hypoglycemia and hypotension was treated with norepinephrine and glucose in fluid therapy. The cat recovered well and was discharged with insulin and amoxicillin-clavulanate. This is the first case of septicemia associated with *Y. pseudotuberculosis* in a cat, suspected of developing the infection after contact with natural reservoirs such as rodents or birds. This route of transmission should be highlighted especially in relation to the zoonotic potential of the bacteria.

## 1 Introduction

*Yersinia pseudotuberculosis* is a gram-negative aerobic or facultative anaerobic rod-shaped bacterium considered as a well-known infectious agent in both humans and animals. It was first described in 1883 (1), and according to the European Center for Disease Prevention and Control (2), is the agent responsible for yersiniosis together with *Yersinia enterocolitica*, the third most prevalent foodborne disease in Europe. In humans, *Y. pseudotuberculosis* infections typically occur by the oro-fecal route, after the ingestion of contaminated food, water, or milk (3) or after contact with animals or their environment (4, 5). Birds and rodents are considered the natural reservoirs (6, 7). In animals, it has been described as an infectious agent in a broad spectrum of species, including pets. In cats, it was first reported in 1967 in the UK (8), but since then, only a few cases of *Y. pseudotuberculosis* infections in cats have been published (9–13). The aim of this case report is to describe for the first time a septicemia associated with *Y. pseudotuberculosis* in a cat presented to an Italian Veterinary University Hospital.

## 2 Case description

**Figure 1:**
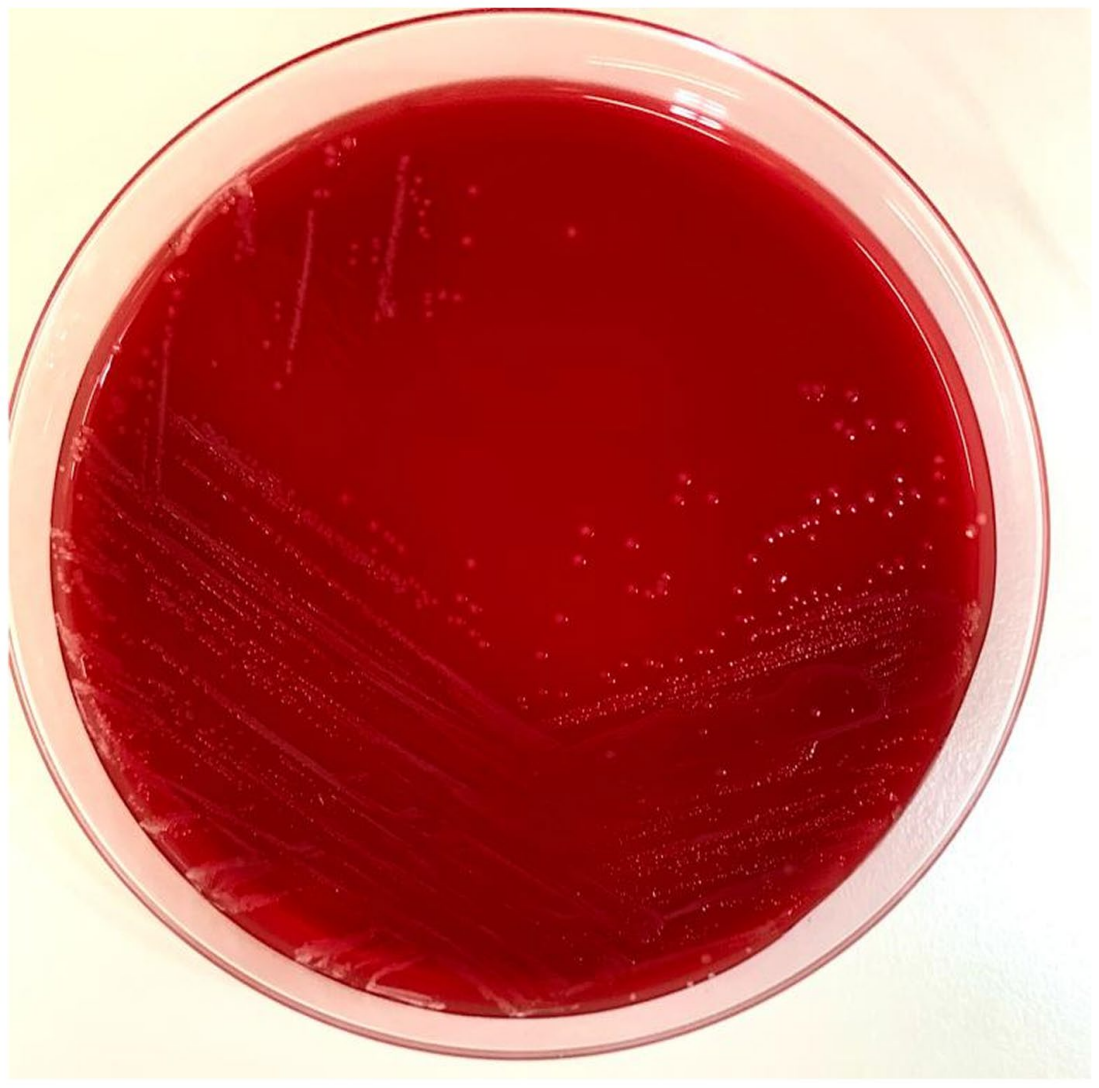
Picture of the Blood Agar with the *Y. pseudotuberculosis* colonies. Ten microliters of the positive blood culture bottle were streaked with a sterile loop and incubated at 37°C for 24 h.

A 14-year-old female spayed domestic short-haired cat was presented at the Veterinary University Hospital at the onset of lethargy and dysorexia in the last few days. The patient was undergoing treatment with insulin (Toujeo 5 UI BID) for a previous diagnosis of acromegaly and diabetes mellitus. Despite the owner continuing to administer insulin, glycemia at home was persistently high (> 500 mg/dL). Before this event, the cat was sporadically seen by the owners hunting wild birds and mice. On physical examination, the cat presented with depression, heart rate of 150 beats/min, blood pressure of 105/76 mmHg, hyperthermia (40.4°C), and clinical signs of dehydration. Hyperglycemia (500 mg/dL) was confirmed in the absence of an increased ketonemia (0.2 mmol/L). Considering liver and kidney function tests, only Alanine Transaminase (ALT) levels were high (280 U/L). According to the primary suspicion of an inflammatory/infectious disease complicating the diabetes mellitus, the patient was hospitalized for proper medical care, and 5 mL of blood, collected by sterile jugular venipuncture, was incubated at 37 ± 1°C in a blood culture bottle (Signal Blood Culture System; Oxoid, Milan, Italy). Replacement fluid therapy was instituted, and once appropriated, a constant rate infusion of regular insulin was administered for glycemic control. Empirical antibacterial treatment with ampicillin-sulbactam (30 mg/kg intravenous TID) was initially prescribed. After 24 h of incubation, the blood culture bottle revealed positivity and was subcultured by streaking 10 microliters in media plates for aerobic (Blood agar with 5% horse blood, MacConkey Agar, Cled Agar), anaerobic (Wilkins-Chalgren Agar, Columbia Agar), and capnophilic (Columbia Agar) bacteria. All the plates were incubated at 37 ± 1°C. At 24 h from subculture, the cultures revealed positivity for isolates in abundant quantity, all in monoculture (Figure 1). One isolate grown from Blood Agar and one from Columbia Agar were subsequently both identified as *Yersinia pseudotuberculosis* through the matrix-assisted laser desorption-ionization time-of-flight mass spectrometry method (MALDI-TOF MS; Biotyper, Bruker Daltonics, Billerica, MA), following the manufacturer’s instructions (Bruker Daltonik, Bremen, Germany), with a score of 2.47 and 2.35, respectively. To confirm the species-level identification, sequencing of the 16S rRNA portion from the extracted DNA was performed, indicating a 99.91% identity with both *Y. pseudotuberculosis* and *Y.pestis.* To rule out *Y.pestis*, an API 20E test (bioMerieux, France) was performed, confirming the suspicion of *Y. pseudotuberculosis* (urease-positive). Subsequent Antimicrobic Susceptibility Testing, performed with the disk diffusion method following CLSI standard and breakpoints (14), showed that the strain was susceptible to every tested antimicrobial (see Table 1). Blood profile was consistent with a septic process, according to leukocytosis (17.160 cells/mm^3^), neutrophilia (14,570/mm^3^) with basophilia and toxic changes, and an increased serum amyloid A concentration (238 μg/mL). However, a clear septic focus was not identified based on clinical and imaging findings from abdominal ultrasonography. Clinical deterioration was recorded on the morning of the second day of hospitalization with the onset of hypotension and hypoglycemia, consistent with a condition of septic shock, and norepinephrine (0.5 mcg/kg/min intravenous) was administered as a first-line vasopressor and fluids were supplemented with glucose to restore arterial blood pressure and normoglycemia, respectively. The cat recovered soon, and it was possible to stop norepinephrine infusion in the evening. General conditions progressively and significantly improved in the next days and the cat was discharged after 4 days of hospitalization with insulin 5 UI BID and amoxicillin-clavulanate 15 mg/kg SID for 10 days. The follow-up controls revealed good general health conditions and antimicrobial treatment was stopped after 10 days.

**Table 1:** Antimicrobial susceptibility testing for *Yersinia pseudotuberculosis* from blood culture.

Antimicrobial	Concentration	Resistance breakpoint (mm)	Observed diameter (mm)	Interpretation
Amikacin	30 mg	≤ 14	22	S
Gentamicin	10 mg	≤ 12	20	S
Streptomycin	10 mg	≤ 11	16	S
Ampicillin	10 mg	≤ 13	30	S
Amoxicillin-clavulanate	20/10 mg	≤ 13	32	S
Piperacillin-tazobactam	110 mg	≤ 17	32	S
Cefazolin	30 mg	≤ 19	30	S
Ceftiofur	30 mg	≤ 17	31	S
Tetracycline	30 mg	≤ 11	29	S
Enrofloxacin	5 mg	≤ 16	30	S
Trimethoprim-sulfamethoxazole	1.25/23.75 mg	≤ 10	28	S

S, Susceptible.

## 3 Discussion

From an evolutionary point of view, *Y. pseudotuberculosis* diverged from a common ancestor with *Y.enterocolitica* approximately 41–186 million years ago (15). The other human pathogen, *Yersinia pestis,* is genetically extremely similar to *Y. pseudotuberculosis* to the point that taxonomically they should be grouped into a single species (16). Furthermore, *Y. pseudotuberculosis* can be subdivided considering the basis of lipopolysaccharide O-side chain into 15 O-serotypes: in Europe, the most frequent serotypes are O:1-O:3 (16). The recent analysis described other two bacterial populations as a part of the “*Y. pseudotuberculosis* complex”: *Yersinia similis* and the “Korean group.” The latter, composed of non-pathogenic strains mostly found in the Korean region (17), has been a matter of debate since Savin et al. (18) proposed to rename it as a novel species, *Yersinia wautersii*, but Neubauer and Sprague (19) argued that it should continue to be considered as a subgroup of *Y.pseudotuberculosis*. On the other hand, *Y.similis* is a non-pathogenic species first identified in 2008 (20), phenotypically indistinguishable from *Y. pseudotuberculosis* with methods such as MALDI-TOF. In this paper, we used genotypic and phenotypic methods to distinguish between *Y. pseudotuberculosis in sensu stricto* and the other members of the *Y. pseudotuberculosis* complex.

In humans, *Y. pseudotuberculosis* not only causes normally self-limiting gastroenteritis but also pseudoappendicitis, arthritis, pharyngitis, and erythema nodosum (21–23). It can also cause bacteremia and sepsis, with a mortality rate as high as 75% (24). Bacteremia cases more commonly involve patients with immunodeficiency (25, 26), but it has also been reported by Hashimoto et al. (27) as a cause of septic shock in an immunocompetent adult. Its zoonotic potential is well-known (2). Fukushima et al. (22) related the presence of a cat-contaminated environment with the onset of fever and diarrhea in two children with fecal culture positive for *Y. pseudotuberculosis* that drunk from a puddle near the cat’s litter. In animals, it has been reported as a cause of enteritis (28) and mastitis in cattle (29), ocular disease in goats (30), and septicemia in beavers (31), and it has been related to multiple deaths in zoological collections (32, 33), including mammals like monkeys, meerkats, and paca (34–36), as well as felids such as lions (37). In cats, only a few cases of *Y. pseudotuberculosis* infections were published (8–13), and there were no reports of isolation from blood. The main symptoms described were lethargy, reduced appetite, and abdominal pain. It has also been described by Thompson (12) and Iannibelli et al. (13) as a cause of hepatitis and enteritis in cats that may become infected by consuming wild animals like rodents and birds. To our knowledge, this is the first report of *Y. pseudotuberculosis* associated with septicemia in a cat. Although the possibility of contamination of the intravenous catheter cannot be excluded, the onset of clinical symptoms such as hyperthermia, leukocytosis, and the development of septic shock suggested that the positive blood culture reflected a real ongoing infection that was successfully treated. Indeed, the patient’s hunting behaviors described by the owners made it likely to have contact with potential reservoirs such as birds or rodents. Furthermore, diabetes mellitus and acromegaly might have predisposed the cat to infection. In contrast with the case reported by Thompson (12), in this case, no specific neurological signs were observed, suggesting that the infection did not spread to the nervous system. In that case, neurological signs in the absence of hematogenous diffusion suggested they were most likely the result of hepatic encephalopathy, and treatment with a combination of marbofloxacin and amoxicillin-clavulanate was successful. Further investigation about the infection route was complicated to realize, but it can be assumed that pathogen transmission probably occurred from the gastrointestinal tract to the bloodstream through translocation, with possible involvement of the liver through the portal system or by ascending the biliary route.

In conclusion, this case report describes septicemia associated with *Y. pseudotuberculosis* in a cat, with a positive outcome reached with amoxicillin-clavulanate treatment based on AST results. Cats with external access showing hunting behavior should be monitored for the risk of contracting bacteria such as *Y.pseuduberculosis* from preys considered to be natural reservoirs, like rodents or birds. This should be considered in relation to the zoonotic potential of the bacteria, especially in situations in which the cat shares the same household with the owners.

## Data availability statement

The original contributions presented in the study are included in the article/supplementary material, further inquiries can be directed to the corresponding author.

## Ethics statement

Ethical approval was not required for the studies involving animals in accordance with the local legislation and institutional requirements because the data used for this study were extrapolated from the clinical data of the patient. Written informed consent was obtained from the owners for the participation of their animals in this study. Written informed consent was obtained from the participant/patient(s) for the publication of this case report.

## Author contributions

RS: Conceptualization, Data curation, Writing – original draft. MG: Writing – original draft, Writing – review & editing. CB: Writing – review & editing. EM: Writing – review & editing. EE: Writing – review & editing. GA: Writing – review & editing. SP: Writing – original draft, Writing – review & editing.
